# Comorbidities and associated factors of painful temporomandibular disorder symptoms in the BigMouth Dental Data Repository

**DOI:** 10.1097/PR9.0000000000001492

**Published:** 2026-08-05

**Authors:** Caroline M. Sawicki, Linda Sangalli

**Affiliations:** a Adams School of Dentistry, University of North Carolina, Chapel Hill, NC, USA; b College of Dental Medicine, Midwestern University, Downers Grove, IL, USA

**Keywords:** BigMouth, Temporomandibular disorders, Comorbidities, Large database

## Abstract

**Introduction/Objectives:**

Temporomandibular disorders (TMD) are associated with multiple comorbidities; however, specific comorbidities may be differentially associated with painful and nonpainful TMD. We describe the prevalence of TMD symptoms, quantified associated comorbidities, and identified associated factors of painful TMD symptoms in a large database.

**Methods:**

Data were extracted from BigMouth repository (January 2014–May 2025) for adults with responses to TMD-related items of “pain upon chewing/talking/jaw use” (used to classify patients into self-reported “painful” vs “nonpainful TMD symptoms”), “jaw popping/clicking,” and “jaw locking.” Associations between painful TMD and comorbidities were calculated with χ^2^ tests and prevalence ratios (PRs). Logistic regression identified factors associated with painful TMD symptoms.

**Results:**

Of 259,768 patients (46.5 ± 17.8 years old, 51.4% women), common symptoms were tooth grinding (21.3%), headaches (23.3%), pain on chewing (9.5%), temporomandibular joint noises (21.6%), and jaw locking (3.1%). Compared with those with nonpainful TMD, those with self-reported painful TMD symptoms had significantly higher prevalence of headaches (38.8% vs 16.1%, PR = 2.41, 95% confidence interval [CI] 2.36–2.46), arthritis (19.4% vs 16.1%, PR = 1.20, 95% CI 1.15–1.26), depression (34.7% vs 18.1%, PR = 1.91, 95% CI 1.84–1.98), anxiety (29.9% vs 21.8%, PR = 1.37, 95% CI 1.31–1.43), sleep apnea (17.7% vs 9.1%, PR = 1.95, 95% CI 1.86–2.05), and gastrointestinal issues (16.4% vs 9.8%, PR = 1.67, 95% CI 1.58–1.77, all *P*'s < 0.001). Each additional somatic comorbidity more than doubled the odds of having painful TMD (*P* = 0.010, odds ratio = 2.67, 95% CI 1.26–5.63); age and sex were not associated.

**Conclusion:**

Painful TMD is strongly associated with psychological and somatic comorbidities. Accumulation of somatic comorbidities increases TMD risk, highlighting the need of integrated medical-dental approaches to TMD care.

## Abstract 2

Supplemental Digital Content is Available in the Text.

Self-reported painful temporomandibular disorder symptomatology is strongly associated with both somatic and psychological comorbidities, with the accumulation of somatic conditions increasing the odds of painful temporomandibular disorder symptoms.

## 1. Introduction

Temporomandibular disorders (TMD) comprise heterogeneous conditions involving pain and/or dysfunction of the masticatory muscles, temporomandibular joints (TMJs), and associated structures.^32^ While TMD ranks as the second most prevalent musculoskeletal condition after low back pain,^44^ with a global prevalence exceeding 30% among adults,^63^ clinically relevant painful TMD affects approximately 10% of the population.^26,49^ TMD imposes a substantial societal and economic burden, with annual US healthcare expenditures estimated at $4 billion, nearly double that of individuals without TMD.^39,44,45,70^ Much of this financial burden is concentrated among a small subset of patients, suggesting important heterogeneity across TMD subtypes.^44^ TMD is also associated with lower quality of life,^48^ greater psychological distress,^53^ and functional limitations.^29^

Multiple comorbidities frequently coexist with TMD, many of which fall under the umbrella of chronic overlapping pain conditions (COPCs).^7,9^ Evidence from cross-sectional and case–control studies shows that adults with TMD exhibit a higher prevalence of migraine,^24,25,33^ headache,^19,24,25^ fibromyalgia,^27,33^ gastrointestinal (GI) disorders,^1,18,27,33^ chronic fatigue syndrome,^1,52^ interstitial cystitis,^10,33^ among others,^18,19^ compared with those without TMD. Adults with TMD present with significantly higher psychological distress,^53^ including anxiety,^22^ depression,^4,22^ and pain catastrophizing.^22^ Higher number of comorbidities has also been correlated with greater TMD pain intensity and chronicity.^17^ Multiple comorbidities also increase TMD risk. For example, findings from the OPPERA study and others indicate that individuals with widespread pain,^28^ multiple painful comorbidities, nonspecific orofacial symptoms,^6^ and psychosocial distress^21^ are at higher risk of developing TMD than those without such conditions. Moreover, individuals with TMD have 6-fold higher odds of developing additional medical comorbidities after TMD onset.^27^

Despite these associations, different TMD subtypes may exhibit distinct comorbidity patterns. To date, few studies have investigated comorbidity patterns by subtype, and most have focused on painful TMD. For example, Dahan et al.^16^ reported significantly higher prevalence of self-reported migraine and chronic fatigue syndrome among individuals with myofascial TMD compared with those without myofascial pain. Similarly, in patients with muscular TMD subtypes, diagnoses of migraine and chronic fatigue syndrome were associated with higher pain intensity and duration, an effect not observed in articular TMD.^17^ Gonçalves et al.^25^ observed increased odds of headache in painful myofascial TMD but not in articular subtypes. However, these studies were limited by small sample sizes and specific clinical populations.

To address these methodological shortcomings, the Consortium for Oral Health Research and Informatics (COHRI) established the BigMouth Dental Data Repository, a national database including medical and dental records from more than 4.5 million individuals across 11 US dental institutions.^61,66,67^ This multi-institutional resource enables large symptom-based epidemiological analyses of TMD and comorbidities, improving external validity of previous findings.^12^

The objective of this study was to characterize comorbidity patterns in adults with self-reported TMD symptomatology. The primary aim was to compare the prevalence and burden of comorbidities between individuals with and without self-reported painful TMD symptoms. The secondary aim was to evaluate comorbidity phenotypes across symptom-based subgroups of painful TMD. The tertiary aim was to identify demographic and comorbidity factors associated with self-reported painful TMD symptoms. It was hypothesized that adults with self-reported painful TMD symptoms would present with a significantly greater comorbidity burden compared with those without painful TMD symptoms, that co-occurrence of multiple TMD symptoms would correspond to increased comorbidity burden, and that selected comorbidities and demographic characteristics would be associated with the presence of self-reported painful TMD symptoms.

## 2. Methods

This study followed the Strengthening the Reporting of Observational Studies in Epidemiology (STROBE) reporting guidelines.^65^

### 2.1. Study design

This cross-sectional study used data extracted from an existing deidentified patient-level database,^54^ declared exempt from further review by the Midwestern University IRB (#IRB-25-0282, June 24, 2025) and approved by BigMouth COHRI clinical review committee.^61^

### 2.2. Setting

The BigMouth Dental Data Repository collects demographic, dental, and medical information from more than 4.5 million patients across 11 participating US dental institutions, members of the COHRI.^8,66,67^ Electronic health records are imported through axiUm software (axiUm Academic Dental Software, Exan Software, Henry Schein One, Melville, NY) and subsequently integrated into the BigMouth database.^43^ Data were extracted from the BigMouth Repository from January 1, 2014, to May 05, 2025, according to eligibility criteria.

### 2.3. Eligibility criteria

Eligible patients were adults with data available to any of the 3 items investigating TMD symptoms (see *Variables and data sources*). Patients with a diagnosis of cancer were excluded to minimize the confounding from complex medical and treatment-related factors. Syndromic patients were also excluded due to potential confounding effects on craniofacial development and pain perception. Finally, children (younger than 18 years) were excluded given the significant difference in comorbidities between the adult and pediatric populations. Pediatric findings using the same BigMouth data set and query have been reported separately.^56^

### 2.4. Variables and data sources

For all eligible patients, the following variables were extracted:

*Demographics* included sex (female, male) and age (years).

*Self-reported TMD symptoms* were obtained from the dental history completed during the patients' first visit by answering the following 3 items: “difficulties and/or pain upon chewing, talking, or using the jaw” (which identified self-reported pain on jaw function); “popping, clicking, or other noises from the jaw” (which identified self-reported joint noises); and “locking of the jaw” (which identified self-reported jaw locking). Patients were included in the analytic cohort if they provided responses to TMD-related items. An additional item (“clenching, bruxism, or grinding”) was obtained from the self-reported dental history; however, this item was not used to define TMD status, as it reflects an oral behavior and not core TMD symptomatology. These variables do not reflect standardized criteria, symptoms frequency, or validated screening instruments.

Type and number of *medical comorbidities* were collected from the patient's medical history recorded at the initial visit in the axiUm electronic dental record. These variables are captured as categorical (present/absent) entries based on patient-reported and/or documented medical history and grouped into predefined categories within the database, including musculoskeletal disorders (eg, arthritis, osteoarthritis, rheumatoid arthritis [RA]), respiratory disorders (eg, sleep apnea), GI conditions (eg, general GI issues, irritable bowel syndrome [IBS]), and neurological/psychological conditions (eg, migraine, headache, self-reported anxiety, and depression). To summarize the overall health burden, 2 indices were created: a psychological comorbidity index (calculated by summing the total number of psychological conditions) and a somatic comorbidity index (calculated by summing the total number of systemic somatic conditions). Details on the specific comorbidities included within each index are provided in Supplemental File (available at https://links.lww.com/PR9/A442).

### 2.5. Bias

To explore a possible selection bias of the patients with available data on TMD-related items, a sensitivity analysis was conducted to compare the included analytic cohort with patients without data on TMD-related items in demographics and comorbidity burden.

### 2.6. Study size

Previous studies have reported moderate-to-large associations between TMD and systemic comorbidities, with effect sizes ranging between Cohen *d* = 0.5 and 0.8.^30^ Research examining associations between TMD and psychological distress has found odds ratios (ORs) of 2.5 to 4.0,^3,62^ while investigations of TMD and headache/migraine have reported ORs of 2.0 to 3.5.^3^ Sample size calculations in these contexts typically require between 100 and 1,000 to detect such effects with 80% power at α = 0.05.^24^ In this study, the BigMouth database large sample size ensures adequate statistical power to detect associations of the magnitude reported in prior work, even after stratifying patients by TMD subtype and comorbidities.

### 2.7. Statistical methods

Data were examined for completeness, and missing data were handled by case-wise deletion.

To test our primary aim, patients with available data to TMD-related items were classified into a self-reported painful TMD symptom group (in case of a positive answer to the item “difficulty and/or pain upon chewing, talking, or using the jaw”) or a nonpainful TMD symptom group (in case of a negative to the same item). The 2 groups were compared in prevalence (frequency distribution) and burden (index) of somatic and psychological comorbidities with Pearson χ^2^ tests and independent *t* tests. Effect sizes were expressed using Phi (φ) coefficients, Cramer *V* (*V*), or Cohen *d* (*d*) and interpret using conventional thresholds: Cohen *d* was interpreted as small (0.20), moderate (0.50), and large (0.80); φ coefficients and Cramer *V* were interpreted as small (0.10), moderate (0.30), and large (0.50) associations.^15^ Prevalence ratios (PRs) and 95% confidence intervals (CIs) were computed using risk estimates from 2 × 2 contingency tables. Sex-based analyses were repeated by comparing the 2 groups only among women and only among men.

To test our secondary aim, patients reporting painful TMD symptoms were further stratified according to the presence of additional self-reported TMD-related symptom domains. This grouping approach was used because the available EHR data did not allow classification according to TMD diagnostic criteria. Thus, symptom-based domains captured in routine clinical intake were used as pragmatic indicators of increasing symptom complexity. Three mutually exclusive groups were created: (1) painful TMD symptoms only (positive answer to “difficulty and/or pain upon chewing, talking, or using the jaw”) and negative responses to joint noises and jaw locking; (2) painful TMD symptoms with joint-related noises (positive responses to painful TMD symptoms and “popping, clicking, or other noises from the jaw,” but negative response to jaw locking); and (3) co-occurring painful TMD, joint-related noises, and functional limitation (positive responses to painful TMD symptoms, joint noises, and jaw locking). These groupings were intended to reflect increasing symptom burden across domains, rather than diagnostic subtypes. Difference in prevalence of comorbidities across groups were evaluated with Pearson χ^2^ tests. Given the known demographic-based difference in comorbidities,^13,34,47^ the comparisons across the 3 groups were repeated by controlling for sex and age with binary logistic regression analyses.

To test our tertiary aim, a multivariable logistic regression analysis was performed to examine associations between comorbidity burden and self-reported painful TMD. Painful TMD was entered as dependent variable, while somatic and psychological comorbidity indexes, presence of intracapsular interference, and demographic factors (age and sex) were independent variables. Adjusted ORs (aORs) and 95% CI were calculated to estimate the strength of associations.

Data were analyzed with SPSS (IBM SPSS, version 31, IBM Corp, Armonk, NY), with *P* values for each test set by Holm–Bonferroni correction for multiple comparison.

## 3. Results

### 3.1. Participants

**Figure 1.**
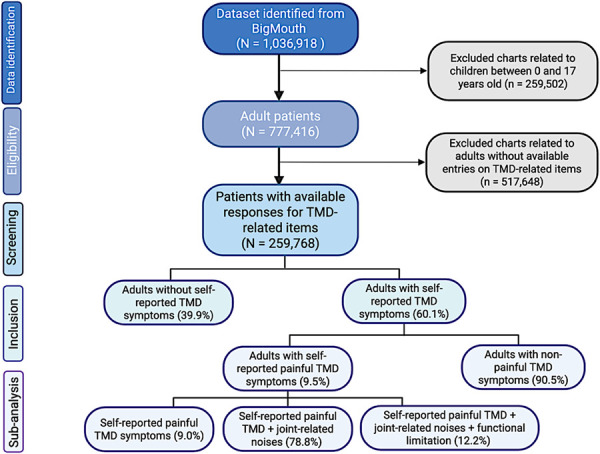
Flowchart of patient selection. TMD, temporomandibular disorder.

The database included 777,416 adult patients (75.0%, mean age 44.5 ± 15.6 years, 54.3% women). Responses to TMD-related items were available for 259,768 adults (33.4%, mean age 46.5 ± 17.8 years, 51.4% women, Fig. 1). A sensitivity analysis was performed to compare patients with and without responses with TMD items within the BigMouth data depository (Table 1). Among those with responses to the TMD-related items, most common TMD signs and symptoms were headaches (23.3%), TMJ noises (21.6%), teeth grinding (21.3%), pain on chewing (9.5%), and difficulty with opening, closing, or locking (3.1%).

**Table 1:** Sensitivity analysis between the selected cohort of patients (ie, those with responses to temporomandibular disorder-related items) and the remaining patients from BigMouth (ie, those without responses to temporomandibular disorder-related items).

Variables	Patients without TMD-related items (N = 517,648)	Patients with TMD-related items (N = 259,768)	P* (effect size)
Demographics			
Age (y), mean ± SD	43.5 ± 17.4	46.4 ± 17.7	**<0.001** (0.17)
Female, n (%)	55.8	51.4	**<0.001** (0.04)
Somatic comorbidities, %			
Headache	12.5	18.2	**<0.001** (0.04)
Migraine	9.2	11.6	**<0.001** (0.04)
Arthritis	20.2	17.9	**<0.001** (0.03)
RA	2.8	2.3	0.008 (0.02)
Osteoarthritis	6.4	59.1	**<0.001** (0.56)
Sleep apnea	13.3	10.1	**<0.001** (0.04)
GI disorders	10.5	10.8	0.102 (0.01)
IBS	0.1	0.7	**<0.001** (0.03)
Psychological comorbidities, %			
Anxiety symptomatology	11.3	22.3	**<0.001** (0.13)
Depression symptomatology	10.6	19.3	**<0.001** (0.11)

Significance is denoted by bolded font.

* Results of χ^2^ tests or independent *t* tests.

GI, gastrointestinal; IBS, irritable bowel syndrome; RA, rheumatoid arthritis; TMD, temporomandibular disorder.

### 3.2. Painful vs nonpainful self-reported temporomandibular disorder symptoms

**Figure 2.**
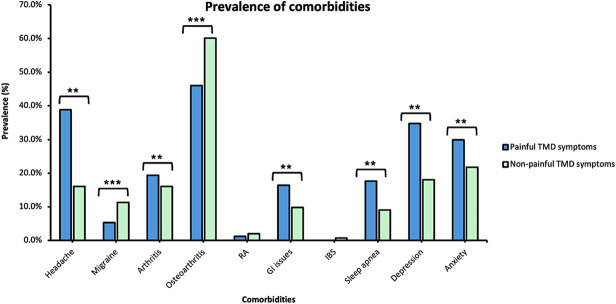
Difference in prevalence of comorbidities among self-reported painful vs nonpainful TMD symptoms. Note: *P < 0.0.05; **P < 0.01; ***P < 0.001. GI, gastrointestinal; IBS, irritable bowel syndrome; RA, rheumatoid arthritis; TMD, temporomandibular disorder.

Compared with those with nonpainful TMD, adults with self-reported painful TMD symptoms had greater somatic comorbidity index (1.6 ± 1.5 vs 0.8 ± 1.1, *P* = 0.013, Cohen *d* = 0.67) and psychological comorbidity index (0.7 ± 1.0 vs 0.4 ± 0.8, *P* < 0.001, Cohen *d* = 0.42). Figure 2 displays differences in prevalence of comorbidities between the 2 groups. Specifically, adults with self-reported painful TMD symptoms presented with significantly higher prevalence of headaches (38.8% vs 16.1%, *P* < 0.001, φ = 0.16, PR = 2.41, 95% CI 2.36–2.46), arthritis (19.4% vs 16.1%, *P* < 0.001, φ = 0.02, PR = 1.20, 95% CI 1.15–1.26), sleep apnea (17.7% vs 9.1%, *P* < 0.001, φ = 0.08, PR = 1.95, 95% CI 1.86–2.05), GI issues (16.4% vs 9.8%, *P* < 0.001, φ = 0.07, PR = 1.67, 95% CI 1.58–1.77), self-reported depression (34.7% vs 18.1%, *P* < 0.001, φ = 0.10, PR = 1.91, 95% CI 1.84–1.98), and anxiety symptomatology (29.9% vs 21.8%, *P* < 0.001, φ = 0.05, PR = 1.37, 95% CI 1.31–1.43), compared with those with nonpainful TMD. Women were significantly more likely to present with self-reported painful TMD symptoms compared with men (52.7% vs 51.0%, *P* < 0.001), although the magnitude of this association was negligible (φ = 0.01, PR = 1.03, 95% CI 1.02–1.05).

Conversely, adults with self-reported painful TMD were less likely to report osteoarthritis (46.0% vs 60.1%, *P* < 0.0001, φ = 0.08, PR = 0.77, 95% CI 0.69–0.84) and migraine (5.3% vs 11.3%, *P* < 0.0001, φ = 0.04, PR = 0.47, 95% CI 0.41–0.54) in their medical history compared with those with nonpainful TMD symptoms. No difference was seen in the prevalence of RA (*P* = 0.185) and IBS (*P* = 0.055) across groups. Significant differences in prevalence of comorbidities were maintained in sex-based analyses (Table 2).

**Table 2:** Sex-based differences in prevalence of comorbidities among adults with and without painful temporomandibular disorder.

Comorbidity	Males (48.6%)		Females (51.4%)		P* (effect size)	PR (95% CI)	*P*† (effect size)	PR (95% CI)
	Nonpainful TMD symptoms (90.8%)	Painful TMD symptoms (9.2%)	Nonpainful TMD symptoms (90.2%)	Painful TMD symptoms (9.8%)				
Somatic comorbidities, %								
Headache	11.4	33.3	20.5	44.1	**<0.001** (0.18)	2.89 (2.79–3.00)	**<0.001** (0.16)	2.15 (2.09–2.20)
Migraine	7.2	3.3	15.1	6.8	**<0.001** (0.03)	0.36 (0.28–0.47)	**<0.001** (0.05)	0.45 (0.38–0.53)
Arthritis	13.8	15.7	18.3	22.3	**0.001** (0.01)	1.14 (1.05–1.23)	**<0.001** (0.03)	1.22 (1.15–1.29)
Osteoarthritis	55.1	44.1	62.9	46.6	0.005 (0.06)	0.80 (0.68–0.95)	**<0.001** (0.09)	0.74 (0.65–0.84)
RA	1.3	1.0	2.8	1.3	0.730 (0.00)	0.78 (0.19–3.16)	0.121 (0.02)	0.47 (0.17–1.26)
Sleep apnea	10.9	18.9	7.2	16.7	**<0.001** (0.06)	1.73 (1.62–1.86)	**<0.001** (0.09)	2.31 (2.16–2.48)
GI disorders	9.9	16.2	9.8	16.7	**<0.001** (0.07)	1.63 (1.50–1.78)	**<0.001** (0.08)	1.71 (1.58–1.90)
IBS	0.6	0.0	0.8	0.0	0.268 (0.01)	0.99 (0.99–1.00)	0.141 (0.01)	0.99 (0.99–0.99)
Psychological comorbidities, %								
Self-reported anxiety symptoms	16.9	25.5	26.49	33.1	**<0.001** (0.05)	1.51 (1.41–1.63)	**<0.001** (0.04)	1.25 (1.19–1.32)
Self-reported depression symptoms	15.4	31.4	20.8	37.1	**<0.001** (0.10)	2.03 (1.91–2.16)	**<0.001** (0.10)	1.78 (1.70–1.86)

Significance is denoted by bolded font.

* Results of χ^2^ tests among males.

† Results of χ^2^ tests among females.

CI, confidence interval; GI, gastrointestinal; IBS, irritable bowel syndrome; PR, prevalence ratio; RA, rheumatoid arthritis; TMD, temporomandibular disorder.

### 3.3. Symptom-based subgroups of painful temporomandibular disorder

Of those reporting self-reported painful TMD symptoms, most adults also reported co-occurring joint-related noises (78.8%), followed by those reporting the co-occurrence of pain, joint-related noises, and functional limitation (12.2%), and those reporting painful TMD symptoms without additional symptom domains (9.0%). Adults with co-occurring painful TMD symptoms, joint-related noises, and functional limitation showed a significantly greater burden of comorbidities, including headaches (*P* < 0.001, *V* = 0.13), migraine (*P* = 0.018, *V* = 0.09), arthritis (*P* < 0.001, *V* = 0.24), and self-reported anxiety symptomatology (*P* < 0.001, *V* = 0.16) compared with the other groups. Those reporting both painful TMD symptoms and joint-related noises (without functional limitation) were significantly more likely to report feelings of depression (*P* < 0.001, *V* = 0.14) and sleep apnea (*P* < 0.001, *V* = 0.12) compared with the other groups. As those with co-occurring symptom domains were more likely to be women (*P* < 0.001, *V* = 0.10) and those reporting painful TMD symptoms only were, on average, 3 to 6 years older (*P* < 0.001, η^2^ = 0.02) compared with the other 2 groups, the analyses were repeated controlling for sex and age. After adjustment, all comparisons remained significant except for sleep apnea and arthritis. Table 3 summarizes the comparison of comorbidity burden across symptom-based subgroups of painful TMD.

**Table 3:** Comparison of comorbidity burden across symptom-based subgroup of self-reported painful temporomandibular disorder.

Comorbidity	Self-reported painful TMD symptoms (9.0%)	Self-reported painful TMD symptoms + joint-related noise (78.8%)	Self-reported painful TMD symptoms + joint-related noise functional limitation (12.2%)	P* (effect size)	*P*† (OR, 95% CI)	*P*‡ (OR, 95% CI)
Somatic comorbidities, %						
Headache	36.4	57.9	62.4	**<0.001** (0.13)	**<0.001** (2.76; 2.56–2.98)	**<0.001** (3.09; 2.56–3.73)
Migraine	9.7	6.6	12.4	0.018 (0.08)	**<0.001** (0.42; 0.27–0.68)	**0.041** (0.59; 0.36–0.98)
Arthritis	14.5	56.9	60.5	**<0.001** (0.24)	**<0.001** (0.11; 0.08–0.16)	0.325 (0.83; 0.56–1.21)
Osteoarthritis	41.7	41.8	25.0	0.798 (0.06)	0.416 (2.60; 0.26–25.86)§	0.552 (2.02; 0.20–20.41)§
RA	2.1	0.0	1.5	0.371 (0.09)	0.969 (0.96; 0.10–9.29)§	0.996 (0.00; 0.00, —)§
Sleep apnea	7.8	21.3	17.2	**<0.001** (0.12)	0.740 (0.94; 0.65–1.36)	0.157 (1.33; 0.90–1.98)
GI disorders	15.8	13.0	11.8	0.724 (0.44)	0.804 (1.14; 0.40–3.27)	0.983 (0.99; 0.30–3.28)
Psychological comorbidities, %						
Self-reported anxiety symptoms	16.7	25.9	39.3	**<0.001** (0.16)	**0.033** (0.76; 0.60–0.98)	**<0.001** (0.57; 0.43–0.75)
Self-reported depression symptoms	15.9	36.7	29.6	**<0.001** (0.14)	**0.021** (1.36; 1.05–1.76)	**0.009** (1.45; 1.10–1.91)

IBS was not included in the analysis due to small sample size and unstable estimates.

Significance is denoted by bolded font.

* Results of χ^2^ tests between the 3 subtypes of painful TMD.

† Results of binary logistic regression models controlling for age and sex. Independent variable: Painful TMD (reference) vs Painful TMD + intracapsular interference.

‡ Results of binary logistic regression models controlling for age and sex. Independent variable: Painful TMD (reference) vs Painful TMD + intracapsular interference + functional limitation.

§ Estimates should be interpreted with caution due to small cell counts.

CI, confidence interval; GI, gastrointestinal; IBS, irritable bowel syndrome; OR, odds ratio; RA, rheumatoid arthritis; TMD, temporomandibular disorder.

### 3.4. Associated factors to self-reported painful temporomandibular disorder symptoms

The logistic regression model showed that somatic comorbidities were independently associated with self-reported painful TMD symptoms. Each additional somatic comorbidity was associated with more than a 2-fold increase in the odds of reporting painful TMD symptoms (*P* = 0.010, aOR = 2.67, 95% CI 1.26–5.63). The presence of intracapsular interference was also associated with higher odds of reporting painful TMD symptoms (*P* = 0.047, aOR = 5.50, 95% CI 1.03–29.47). Neither sex (*P* = 0.337) nor age (*P* = 0.247) were significantly associated. Although adults with psychological comorbidities showed higher odds of self-reported painful TMD symptoms (aOR = 1.58, 95% CI 0.59–4.26), the association was not statistically significant (*P* = 0.366).

## 4. Discussion

This large, multi-institutional BigMouth Dental Data Repository study aimed to characterize comorbidity patterns among adults with symptom-based subgroups of painful TMD and identify factors associated with self-reported painful TMD symptoms. Individuals with self-reported painful TMD symptoms exhibited significantly higher somatic and psychological comorbidity burden than those with nonpainful TMD. Among subgroups, those with co-occurring painful TMD symptoms, joint-related noises, and functional limitation demonstrated significantly greater comorbidity burden. Each additional somatic comorbidity was associated with more than doubled the odds of self-reported painful TMD symptoms, evidencing a moderate association between systemic health and TMD pain expression.

### 4.1. Individuals with painful temporomandibular disorder symptoms exhibited higher and specific comorbidities compared with those without painful temporomandibular disorder

Approximately 1 in 10 individuals with TMD symptoms reported painful symptomatology. Given the lack of diagnostic data, the self-reported painful TMD group may have included both myogenous and articular pain sources. Conversely, the nonpainful group included individuals reporting asymptomatic TMJ noises and/or jaw locking. Accordingly, findings should be interpreted as reflecting a symptom-defined cohort rather than clinically confirmed TMD diagnoses.

Compared with those with nonpainful TMD symptomatology, individuals with self-reported painful TMD symptoms exhibited higher prevalence of several comorbidities. Headache was more than twice as common, self-reported depression symptomatology nearly doubled, and self-reported anxiety symptoms increased by about one-third. Sleep apnea, GI complaints, and arthritis were also more prevalent. By contrast, individuals with non-painful TMD were more likely to report migraine and osteoarthritis. Although statistically significant, effect sizes demonstrate small associations for most individual comorbidities (φ = 0.02–0.16), whereas cumulative somatic burden showed a moderate association (effect size = 0.67). The observed association between painful TMD symptoms and cumulative somatic comorbidity burden is consistent with findings from the OPPERA prospective cohort study, where global somatic symptom burden was among the strongest predictors of incident TMD (hazard ratio = 1.33).^21^

These findings align with previous literature. The magnitude of association between painful TMD symptoms and headache in this study (φ = 0.16, PR = 2.41) is comparable with previous epidemiological studies reporting approximately 2- to 5-fold increased odds of headache conditions among individuals with painful TMD.^25,51^ Individuals with myofascial TMD exhibit greater prevalence of self-reported migraine,^16,17,25^ headache,^25^ and chronic fatigue syndrome^16,17^ compared with those without myofascial TMD. The magnitude of association observed for depression and anxiety is consistent with previous studies demonstrating that individuals with painful TMD have ∼1.5- to 4-fold greater odds of reporting psychological distress compared with individuals without TMD, depending on the population and diagnostic criteria used.^21,22^ Although not assessed in this study, other comorbidities (eg, fibromyalgia, chronic fatigue syndrome, and low-back pain) were also significantly more common among patients with TMD with widespread body tenderness than among those without widespread pain.^13^ Similar to our results, no significant differences were reported in IBS prevalence across TMD subtypes^17^ and between TMD with and without widespread pain.^13^ Moreover, number of comorbidities correlated with longer pain duration and, specifically among individuals with myofascial TMD, greater pain intensity.^17^ Similarly, other reports using the Research Diagnostic Criteria for TMD (RDC-TMD) found that patients with myogenous TMD were more likely to report headache, gastric acid reflux, and fibromyalgia, among other nonspecific symptomatology (eg, fainting, dizzy spells, sore throats) compared with those with arthrogenous TMD.^31^ In our sample, we interpret the higher prevalence of migraine among individuals with nonpainful TMD as reflecting self-report misclassification (particularly if participants interpreted migraine as general headache) rather than a true difference between groups. This limitation is consistent with the clinical overlap between migraine and tension-type headache, which self-reported questionnaires often fail to distinguish accurately.

Regarding psychological comorbidities, prior research has consistently shown that adults with muscular TMD experience greater depression,^4,31^ anxiety,^31^ and overall psychological distress^35^ compared with articular TMD. Auerbach et al.^4^ proposed that this pattern may reflect a stronger link between emotional distress and pain of muscular origin. Nonetheless, other studies have found no significant differences in psychological distress across TMD subtypes,^41,50^ suggesting possible population or methodology-dependent variability.

The higher prevalence of systemic and psychological comorbidities among individuals with self-reported painful TMD symptoms may reflect shared mechanisms across chronic pain conditions, including alterations in pain processing, stress responsivity, and behavioral factors.^68,71^ Prior work has shown substantial overlap between TMD and other chronic pain and psychosocial disorders, including headache, anxiety, and depression.^33,60^ These patterns are often discussed in the context of broader pain amplification or dysregulation processes^11,57^; however, the present data set does not include measures of pain processing or experimental pain sensitivity. As such, no mechanistic inferences can be made, and these findings should be interpreted as population-level associations and not evidence of specific underlying biological pathways.

### 4.2. Greater symptom complexity corresponds to higher comorbidity burden in self-reported painful temporomandibular disorder symptoms

Painful TMD symptoms are typically the primary driver of treatment-seeking behaviors and the primary focus of clinical assessment and management, whereas isolated joint noises may not warrant intervention in the absence of pain or functional limitation. In this study, almost 80% of adults with self-reported painful TMD symptoms also reported TMJ noises, while a smaller proportion (12.2%) presented with the full triad of symptoms (ie, pain, self-reported TMJ noises, and jaw locking) or with pain alone without additional symptom domains (9.0%), a distribution that aligns with similar literature.^24^

Individuals reporting co-occurrence of painful TMD symptoms, joint-related noises, and functional limitation were more likely to report multiple comorbidities, including headache, migraine, arthritis, and self-reported anxiety. Although these differences were statistically significant, their magnitude was generally small (effect size: 0.09–0.24), suggesting that no single comorbidity strongly distinguished symptom subgroups. These results align with prior research where the presence of multiple TMD-related symptoms is associated with a higher prevalence of headache and migraine in a dose–response manner.^24^ Other studies similarly reported statistically significant differences in comorbidity burden and psychological functioning between symptom-defined groups, although these observed differences generally reflect overlap instead of complete separation between subgroups.^31^ Hence, these results suggest that the co-occurrence of multiple TMD-related symptom domains may identify individuals with a greater burden of comorbidities. This observation supports the importance of considering the overall complexity of symptom presentation instead of focusing exclusively on pain status. While shared mechanisms such as altered pain processing and central sensitization mechanisms,^13,24^ the present findings should be interpreted within the context of self-reported symptoms. Women were more likely to report co-occurring symptom domains, which aligns with established sex differences in TMD prevalence, complexity of symptom presentation, transition from nonpainful to painful TMD symptoms, and prognosis.^5,37^

### 4.3. Cumulative somatic burden was associated with self-reported painful temporomandibular disorder symptoms

Somatic comorbidity index and intracapsular interference were found to be independently associated with self-reported painful TMD symptoms, consistent with prior literature. The presence and number of comorbid health conditions, among a list of 20 comorbidities not necessarily painful, was found as the strongest predictor of first-onset TMD in the OPPERA study.^6,55,60^ The presence of pain elsewhere in the body was found to consistently predict TMD^6,28^ and TMD chronicity.^17^ In this study, demographic characteristics such as age and sex and psychological comorbidities were not associated with self-reported painful TMD symptoms. Other studies in the literature showed that baseline measures of depression,^59^ affective distress,^21^ health anxiety,^2^ stress,^21,59^ somatic and post-traumatic stress disorder symptoms,^21^ and mood^59^ significantly predicted new-onset TMD and other chronic pain conditions, such as musculoskeletal pain,^42^ low-back pain,^36,42^ and chronic widespread pain.^40^ Nevertheless, this discrepancy may reflect differences in study design, sample size, self-reported symptomatology and medical condition, and target population. Such psychological factors have also been implicated in the maintenance of TMD symptoms,^21^ suggesting that their consideration in TMD care may have direct implication for disease prognosis.

These findings suggest that TMD should not be considered as an isolated condition.^13,64,69^ By contrast, its onset or painful manifestation may be predicted by general indicators of poor systemic health.^60^ Collecting a comprehensive medical history is therefore essential to identify individuals at higher risk for TMD, as their management may be tailored and personalized to achieve more favorable prognosis.^13^

### 4.4. Limitations

As observed in the sensitivity analysis, patients with available responses for TMD-related items were, on average, 3 years older; had 4.4% lower proportion of women; higher prevalence of headache, migraine, osteoarthritis, IBS, anxiety/depression symptoms; and lower prevalence of arthritis, RA, and sleep apnea compared with those without such data. A plausible explanation is that providers may be more likely to collect TMD-related information when patients present with orofacial pain complaints at their initial visit, whereas such questions may be omitted when deemed not clinically relevant. Because the reason for the dental visit was not captured from the available BigMouth data set, these unmeasured factors could introduce potential confounding. Hence, the analytic sample may overrepresent individuals with TMD-related concerns and reflect provider-driven documentation patterns for whom TMD-related assessment was clinically indicated. Despite this selection bias, these differences do not undermine the validity of comparisons between painful and nonpainful TMD symptoms and their associations with somatic and psychological comorbidities. The use of a single self-reported pain item may not fully distinguish TMD-related pain from other sources of orofacial pain (eg, odontogenic pain), which may contribute to misclassification. TMD-related symptoms were recorded as binary variables without information on symptom frequency or duration, limiting comparability with validated screening instruments and potentially introducing additional misclassification.

In the BigMouth repository, comorbidities and TMD symptoms are based on self-reported or documented medical history and not from standardized diagnostic criteria or validated screening instruments. Nevertheless, previous studies have shown that self-reported TMD pain shows good reliability and validity when compared with Diagnostic Criteria for TMD (DC-TMD) standardized clinical assessment.^38,46^ Moreover, the TMD-symptom item questions included in the BigMouth standardized intake questionnaire are drawn from TMD screening recommended by the American Academy of Orofacial Pain.^20^ Similar questions have been used to define TMD cohorts in other large, multicenter studies.^14,58^ Similarly, validation studies have shown acceptable sensitivity and specificity of self-reported medical conditions compared with medical records.^23^ However, future replication studies may benefit from using other nationally representative databases (eg, Epic Cosmos), which integrates dental and medical data within a single EHR system. Given the cross-sectional design, the multivariable analysis reflects associations and not predictive relationship, limiting conclusions about temporality.

Despite these limitations, the study offers several strengths, including a large, multi-institutional sample, sufficient power to examine sex-based differences, and secondary analyses of data routinely collected in clinical settings.

## Disclosures

The authors have no conflicts of interest to declare.

## Supplemental digital content

Supplemental digital content associated with this article can be found online at https://links.lww.com/PR9/A442.
