# Supplementary material for: Comorbidities and associated factors of painful temporomandibular disorder symptoms in the BigMouth Dental Data Repository
Source: Pain Rep. 2026 Aug 5;11(5):e1492. doi: 10.1097/PR9.0000000000001492 (PMC13446968; doi:10.1097/PR9.0000000000001492)
Supplement: Supplementary file 1 [file painreports-11-e1492-s001.pdf]

Supplemental File. Comorbidities and review of systems reported in the medical history.

**Category**	**Symptom / comorbidity**
Musculoskeletal disorders	Arthritis
	Gout
	Lupus
	Osteoarthritis
	Osteoporosis
	Rheumatoid arthritis
Respiratory disorders	Sleep apnea
	Tuberculosis
	Crohns disease
	Sinusitis
	Shortness of breath
	Pneumonia
	Emphysema
	Bronchitis
	Asthma
Gastrointestinal conditions	Heartburn
	Irritable bowel syndrome
	Gastrointestinal disorder
Neurological disorders	Dementia
	Migraines/headaches
	Multiple sclerosis
	Nerve problem
	Parkinson disease
	Post traumatic stress disorder
	Seizure/epilepsy
	Stroke
Psychological conditions	Feelings of anxiety
	Feelings of depression
